# Decision of the Optimal Rank of a Nonnegative Matrix Factorization Model for Gene Expression Data Sets Utilizing the Unit Invariant Knee Method: Development and Evaluation of the Elbow Method for Rank Selection

**DOI:** 10.2196/43665

**Published:** 2023-06-06

**Authors:** Emine Guven

**Affiliations:** 1 Department of Biomedical Engineering Düzce University Düzce Turkey

**Keywords:** gene expression data, nonnegative matrix factorization, rank factorization, optimal rank, unit invariant knee method, elbow method, consensus matrix

## Abstract

**Background:**

There is a great need to develop a computational approach to analyze and exploit the information contained in gene expression data. The recent utilization of nonnegative matrix factorization (NMF) in computational biology has demonstrated the capability to derive essential details from a high amount of data in particular gene expression microarrays. A common problem in NMF is finding the proper number rank (r) of factors of the degraded demonstration, but no agreement exists on which technique is most appropriate to utilize for this purpose. Thus, various techniques have been suggested to select the optimal value of rank factorization (r).

**Objective:**

In this work, a new metric for rank selection is proposed based on the elbow method, which was methodically compared against the cophenetic metric.

**Methods:**

To decide the optimum number rank (r), this study focused on the unit invariant knee (UIK) method of the NMF on gene expression data sets. Since the UIK method requires an extremum distance estimator that is eventually employed for inflection and identification of a knee point, the proposed method finds the first inflection point of the curvature of the residual sum of squares of the proposed algorithms using the UIK method on gene expression data sets as a target matrix.

**Results:**

Computation was conducted for the UIK task using gene expression data of acute lymphoblastic leukemia and acute myeloid leukemia samples. Consequently, the distinct results of NMF were subjected to comparison on different algorithms. The proposed UIK method is easy to perform, fast, free of a priori rank value input, and does not require initial parameters that significantly influence the model’s functionality.

**Conclusions:**

This study demonstrates that the elbow method provides a credible prediction for both gene expression data and for precisely estimating simulated mutational processes data with known dimensions. The proposed UIK method is faster than conventional methods, including metrics utilizing the consensus matrix as a criterion for rank selection, while achieving significantly better computational efficiency without visual inspection on the curvatives. Finally, the suggested rank tuning method based on the elbow method for gene expression data is arguably theoretically superior to the cophenetic measure.

## Introduction

Nonnegative matrix factorization (NMF) algorithms have been advanced for the application fields of bioinformatics, artificial intelligence [1], signal processing systems [2], and music signal processing systems [3]. Lee and Seung [4] formulated a parts-based illustrated algorithm to solve the problem of the NMF puzzle. Furthermore, various algorithms have been established to develop a solution to the NMF problem depending on the field [5-8].

Several approaches have been developed for clustering samples, mutational processes, and gene expression levels that draw similar expression motifs [4,9-11]. However, cancer analysis and classification based on genomic data offers a more powerful method that approach the sensitivity of advanced computational techniques to tackle certain problems such as modeling multiple, heterogeneous populations and reducing the number of variables (genes or mutations). Consequently, the choice of a trivial number of discriminatory features from thousands of features enhances crafting successful pinpointing classification systems [12-14]. Although neural networks are prone to overfitting, if the examined structure is noisy, as in the case of tumor expression profiling [15], Pal et al [12] suggested a variation of a multilayer perceptron network for biomarkers identification. Nevertheless, these approaches have severe constraints in capturing the entire framework essential in the data. Moreover, they generally highlight the dominant forms in a data set and cannot detect different signatures with a universal standard. Thus, an unbiased technique is needed for deciphering many clusters without visual inspection that is also capable of utilizing a computational program.

A common problem in conventional multivariate data analysis methods such as factor analysis (FA), principal component analysis (PCA), cluster analysis, and NMF is to detect the proper number (r) of factors, principal components, clusters, and ranks, respectively. Item redundancy is common in long questionnaires such as those used in a pilot questionnaire study, arguing for the utilization of FA and the variance inflation factor on a lifestyle questionnaire. Staffini et al [16] concluded that both methods are acceptable for item reduction; however, both of these techniques might produce distinct features as an outcome.

The aim of this study was to utilize the unit invariant knee (UIK) method for obtaining related biological and molecular correlations in gene expression data. The UIK method is used to catch compositions essential for the data and to offer biological understanding by systematizing both the features and samples. The approach is based on a “knee point” and its unit invariant estimation using the extremum distance estimator method introduced by Christopoulous [17]. In this regard, NMF decomposes the gene expression data set into fragments of evocative features such as metagene and mutational signatures. When applying this method to conventional factorization techniques such as PCA or FA with World Values Survey Wave 5 United States data [18], certain factors (elements) clearly explained the questionnaire responses (1=“Not at all like me”...6=“Very much like me”) [19,20].

Therefore, given an NMF method and a data set (a target matrix), the tens of thousands of genes regarding a small number of signatures can be analyzed. Gene expression patterns of samples can then be studied to determine the expression motifs of the signatures. The signatures define an interesting decomposition of genes, analogous to the motifs of Hutchins et al [10] in which the first value is selected where the residual sum of squares (RSS) curvature presents an inflection point. The machinery of the UIK method can then be used to detect this inflection and expression motifs define a robust clustering of samples.

In this study, the elbow technique was considered for model selection utilizing alternative parsing and its robustness was evaluated [19,21]. The idea behind this approach is to develop an unbiased computable optimization point of the RSS curve that can then be used to select tuning parameters. The UIK method has proven to be useful for a variety of models, from classifying recordings of echolocation to a decision of predictive models for soil carbon at the field scale [22,23], but has not been used for NMF on genetic data to date. The advantage of the UIK method relative to the cophenetic measure method [24,25], as another NMF rank estimation measure, is that UIK yields a closed-form formula that can provide greater insight and computational speed in simulations, which can then be applied for selecting the rank of NMF for real high-dimensional hyperspectral data.

Finally, this study applies the combination of NMF and the UIK method (designated the uikNMF method) to simplify cancer classification tasks by clustering tumor samples and mutational signature data sets. This enables illustrating numerous sturdy decompositions of genetic and mutational signatures from experimental and simulated data sets.

## Methods

### NMF Approach

Given a target matrix V^m×n^, NMF identifies nonnegative matrices such that N^m×r^ and M^r×n^ (ie, with all entries≥0) to present the matrix decomposition as:

V ≈ NM **(1)**

In practice, N is typically viewed as a basis or metagenes matrix, and the mixture coefficient matrix and metagene expression profiles refer to the matrix N. The rank factorization is chosen such that r≤min(m,n). The goal behind this selection is to explain and split the details classified among V into r factors (ie, the columns of N). Given a matrix V^m×n^, NMF finds two nonnegative matrices, N^m×r^ and M^r×n^ (ie, with all elements≥0), to represent the decomposed matrix as

V ≈ NM,

for instance by natural demanding of nonnegative N and M to minimize the reconstruction error:

||V – NM||_F_, subject to N ≥ 0, M ≥ 0 **(2)**

In this case, we consider a gene expression data set characterized by the expression levels of *m* genes (probes) by *n* samples of unique tissues, cells, cell lines, time points, or experiments. The number *m* of genes usually ranges from hundreds to thousands, and the *n* of experiments or patients is typically 100 for gene expression research. The gene expression data set is presented by a matrix of expression *V* of size N×M, whose rows consist of the expression levels of *m* genes and columns consist of *n* samples.

The aim is to identify a small number of rank factorizations, each defined as a positive linear combination of the *V* target matrix. The positive linear combination of metagenes is described by the gene expression motif of the samples. To obtain a dimensional reduction of the microarray data and evaluate the distinctions among samples, NMF was implemented utilizing R statistical environment version 3.6.3 with the “NMF” package [26].

### Cophenetic Measure

In the framework of classification analyses, Brunet et al [9] suggested utilizing the *cophenetic correlation coefficient* as a metric asset of the clusters. Furthermore, a cophenetic measure was proposed as one of the metrics utilizing the consensus matrix as a criterion for rank selection [25]. Studying the values of the consensus matrix as a similarity metric, the cophenetic correlation coefficient is defined as the correlation between the sample distances induced by the consensus matrix and the cophenetic distances obtained by its hierarchical clustering.

### Proposed UIK Method

Hutchins et al [10] demonstrated how the variation in the RSS of the estimated matrix resulting from NMF analysis reveals a robust approximation of the proper number of elements (r). They employed Lee and Seung’s [4] algorithm to select r, in which the plot of the RSS presents the first inflection point. In practice, the rank factorization r can be computed with a considerably smaller number of iterations, typically 20-30 runs for each value of r. In contrast, an optimal NMF interpretation requires a couple of hundred random restarts, which is computationally costly.

For instance, a fundamental step for any unsupervised algorithm is to determine the optimal number of clusters (k) into which the data may be clustered [27]. The *elbow method* is one of the most popular methods to determine the optimal value of such components of optimum features [17,18]. The utilization of UIK methodology for identification of the knee (elbow) point of a curve has consistently proven to be immensely advantageous in a wide variety of studies to locate the optimal number of “components” on a scree plot of k-means, PCA, FA, and NMF [27-32].

**Figure 1:**
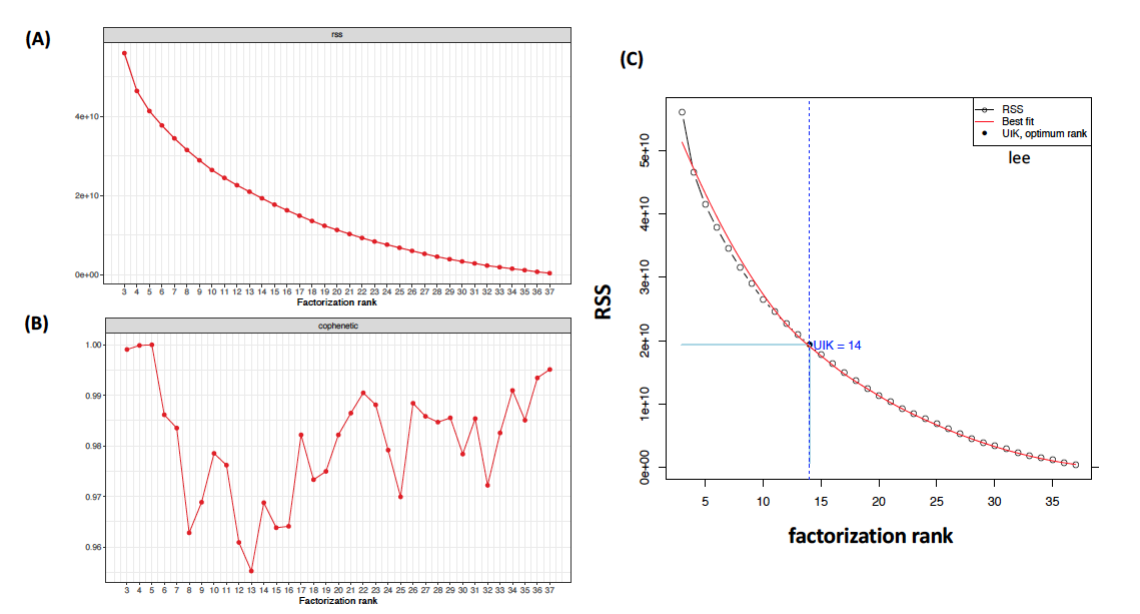
(A) Rank survey plots for residual sum of squares (RSS) and (B) cophenetic coefficient curves factorization rank. The factorization rank ranges from 3 to 37. The aim is to decide whether the optimal rank factorization is very rigid by simple visual inspection. (C) The function of factorization rank is selected as the emergence rank of the RSS survey. The rank range between knee points is detected by the uik() function of the R package "inflection" at the curve of the cumulative rank units. The best fit is determined using a linear regression model.

In many cases, utilization is referred to as uik(x,y), where x is the vector of ranks, components, clusters, or factors and y is the related vector of the RSS curve [10,22,33]. In regression analysis, the term mean squared error (MSE) is sometimes used to refer to the unbiased estimate of error variance (ie, the RSS divided by the degrees of freedom). Ulfarsson and Solo [34] proposed a metric for rank selection in NMF by selecting the tuning parameters of an unbiased computable estimator of the MSE [25]. Thus, as illustrated in Figure 1, the aim is to find an inflection where r meets the proper number of the factorization ranks utilizing the “elbow point,” which is virtually the point where a severely decreasing or increasing curve begins to turn “flat enough” [19,20,22,33,35]. Furthermore, this study considered the function of the rank factorization curve and used the function uik() from the R package *inflection* to select the optimal rank [33,36,37]. The uik() function detects the factorization rank when the curve begins to climb faster (start point) and the point beyond which the curve flattens out (ending point), which are generally known as the *knee points* of a curve (Figure 1). In Figure 1, the emergence of factorization rank for the Golub et al [38,39] gene expression data set is shown on the rank survey plot. The optimal rank of the RSS plot is in between knee points detected by the uik() function of the R package *inflection* at the curve to which the cumulative rank factorization belongs*.*

### Cross-validation

This study used cross-validation to select an optimal number of implicit elements in NMF. The goal of NMF is to obtain low-dimensional N and M with all nonnegative elements by minimizing the reconstruction error |V – NM|^2^. Leaving out a single entry of V (eg, **V_ab_**) and implementing NMF of the resulting matrix may produce a different result than the actual result. In other words, finding N and M while minimizing reconstruction error over all nonmissing entries results in:


∑_ij≠ab_(V_ij_ – [NM]_ij_)^2^
**(3)**

Consequently, the left-out element V*_ab_* can be predicted by calculating [**WH**]_ab_ and then determining the prediction error as:


E(ab) = (V*_ab_* – [**WH**]_ab_)^2^
**(4)**

One can repeat this process by crossing out all entries of V*_ab_* one at a time and adding up the error of prediction overall, *a_a_* and *b_b_*. This will lead to the predicted residual sum of squares (PRESS) value. The PRESS value is defined as E(r) = ∑_ab_E(ab), which will strongly depend on the rank r. The prediction error, E(r), will have a minimum defined as an “optimal rank” r.

Since the NMF must be reiterated for each crossed-out value and might also be difficult to code (depending on the target matrix entries and how smooth it is to implement NMF with missing values), this can be a computationally expensive procedure. For instance, in PCA, one can avoid this by crossing out entire rows of V, which eventually speeds up the computing [40]. All the traditional cross-validation rules can apply here. Therefore, by not including multiple entries instead of a single entry and iterating the computation process by bootstrapping the entries instead of looping over all the entries, both techniques can help speed up the procedure.

Note that various techniques have been developed to select the optimal rank factorization. For example, Brunet et al [9] suggested seizing the first value of r for which the cophenetic coefficient value was decreasing, whereas Frigyesi et al [11] considered the smallest value at which the decrease in the RSS is lower than the decay of the RSS simulated from random data. The aim of this study was to decide how and which approach performs better on an estimation of the latent factors given different algorithms of NMF.

### Gene Expression Data Set

This study illustrates the utilization of NMF based on the UIK method to select the optimal rank on the RSS curve with a leukemia gene expression data set (esGolub) in simplifying cancer subtypes [38,41,42]. This data set has been used in several previous studies on NMF and is built in the NMF package’s data [9,26,43], packed into an ExpressionSet object [39]. To achieve biologically meaningful results, we used the entire gene expression data set including 5000 features for 38 leukemia samples. The difference between acute myelogenous leukemia and acute lymphoblastic leukemia (ALL) has been noted. ALL is also separated into two subtypes: T-cell and B-cell ALL.

Furthermore, this data set has served as a touchstone in cancer classification at the molecule, histology, and stage levels [38,44]. In this study, this data set was reprocessed to compare several clustering techniques regarding their effectiveness and permanence in recuperating other differentially expressed genes (DEGs) and associated pathways. Before the NMF procedure, dimension reduction is recommended for larger gene expression data sets by nonspecific criteria based on the characteristics of the expression estimates (ie, the mean threshold of variance and genes with the smallest average variances) [45].

For example, by looking at the NMF rank survey plot of RSS in Figure 1, we want to decide how many basis vectors we should keep to obtain the optimal rank of the target (original) matrix. To achieve such a task, an unbiased technique for deciding the number of clusters without visual interpretation that is simultaneously capable of utilizing a computational program is needed.

### Simulated Mutational Processes Data

The simulated mutational process data obtained from Alexandrov et al [46] is publicly available as a MATLAB file on SigProfiler [47]. They identified the handful of functional processes for a group of 100 simulated cancer genomes based on the repeatability of their signatures and low error for reconstructing the novel catalogs. The data set was generated by employing 10 mutational processes with different signatures (motifs), each with 96 mutation types, and adding a Poisson noise. The data also correspond to the six subtypes: C:G to A:T, C:G to G:C, C:G to T:A, T:A to A:T, T:A to C:G, and T:A to G:C and their immediate 5′ and 3′ sequence background.

Analyses were performed utilizing the R programming language. Before the procedure, the low-quality genes with an inadequate number of reads were eliminated and gene expression values were converted to a logarithmic scale. The data set (Table 1) was then normalized by computing the averages of each sample in R. The *NMF* R package was used to draw plots of rank surveys using the plot() function [48]. Rank survey analysis was performed to compare the optimal rank with distinct methods using the *inflection* package’s uik() and check_curve() functions [36]. The readMat() function of the R.matlab package [49] was used to import the simulated mutational processes data (Table 1) from the MATLAB file into the R environment (see Supplementary Data S1 in Multimedia Appendix 1).

**Table 1:** Gene expression and simulated mutational data sets.

Data set	Size	Samples
esGolub gene expression	5000×38	38
Mutational processes	100×96	96

## Results

### Applications of NMF Based on the UIK Method

#### Leukemia (esGolub) Data Set

**Figure 2:**
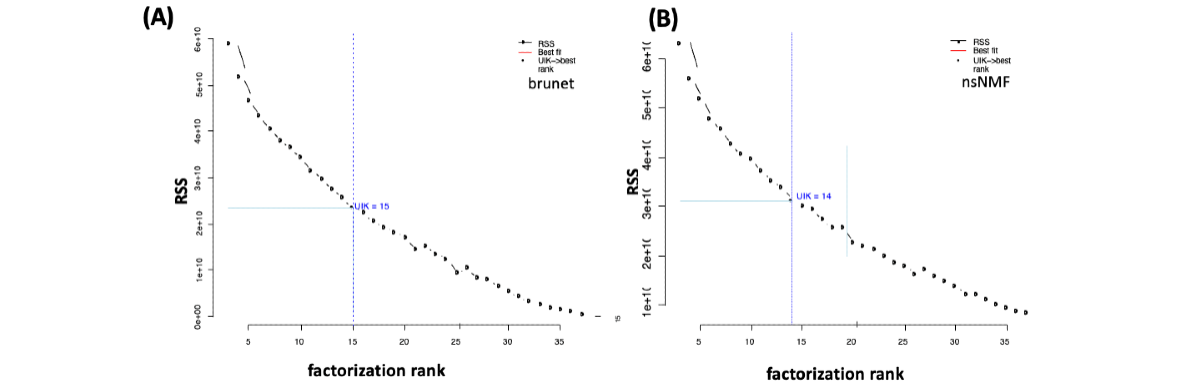
Application of the unit invariant knee (UIK) method on different algorithms: (A) “Brunet” and (B) “nsNMF.” The optimal rank, which UIK represents, is 15 for the Brunet algorithm, whereas the UIK of the nsNMF algorithm reveals 14 as an optimum rank, similar to the “Lee” algorithm.

The present results are based on the NMF package of Gaujoux and Seoighe [26] combined with the technique introduced by Hutchins et al [10] (Figure 1). However, as shown in Figure 2, this study also tested other algorithms taken from the “brunet” and “nsNMF” algorithms to illustrate remarkable differences. It is important to emphasize that there is no remarkable base in the experimental data examined herein. Consequently, it is not possible to demonstrate considerable doubt that the proposed approach operates effectively on the experimental data set. As indicated in Figure 2, the uik() function selects the optimal rank as the curve starts to decline faster (start point) and the point beyond that the curve flattens out (ending point), which are generally known as the knee points of a curve (Figure 1). The UIK method identified 15 components for the brunet algorithm, whereas the nsNMF algorithm detected 14 latent factors as the best representation for the whole esGolub data set.

**Figure 3:**
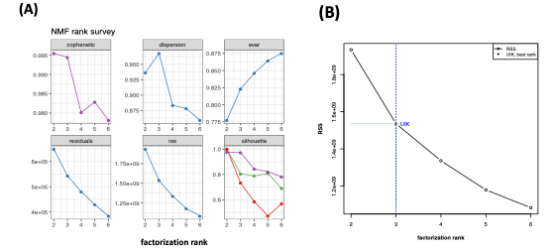
(A) Estimation of the optimal rank. Nonnegative matrix factorization (NMF) survey plot of quality measures obtained from factorization rank from 2 to 6 by running the target matrix esGolub [1:200] 10 times. (B) The function of factorization rank is selected as the emergence rank of the residual sum of squares (RSS) survey. For example, the rank range of 2 to 6 is between knee points detected by the R inflection package's uik()function at 3. Overall, the method of the UIK estimation was confirmed with former results.

By simply looking at the cophenetic correlation or RSS plots of rank factorization in Figure 3A, one can confirm that the optimum rank factorization is 3. For performance reasons, the submatrix esGolub (1:200) was initially performed with only 10 runs for each rank value. As demonstrated in Figure 3B, the UIK method of optimal rank factorization was validated by comparing with Gaujoux’s estimates of the esGolub subdata set [50] (also see Supplementary Data S2 in Multimedia Appendix 1). Consensus methods converged on a rank of 3, replicating the result of Brunet et al [9], in which it was proposed that 3 factors yielded a more complete understanding of the esGolub data set with 200 features from 38 leukemia samples.

#### Simulated Mutational Process Data

**Figure 4:**
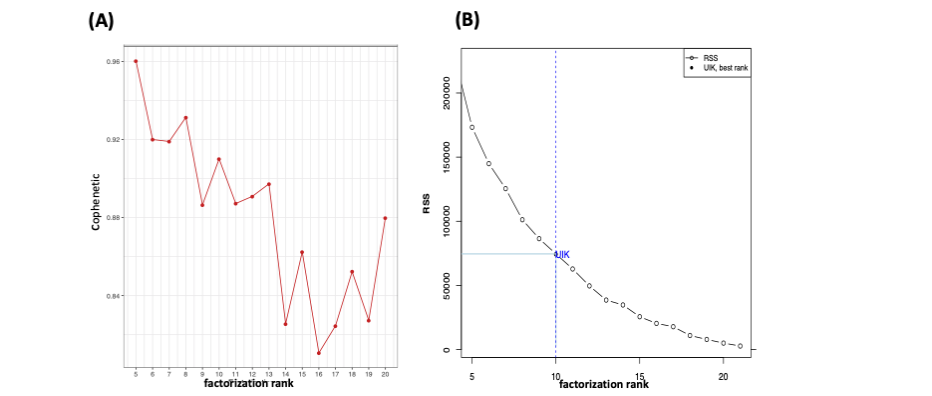
(A) It is complicated to locate the optimal rank with the cophenetic correlation coefficient approach. (B) However, the unit invariant knee (UIK) method can facilitate this decision more quickly and more accurately, which agrees with the number of signatures detected by Alexandrov et al [46]. RSS: residual sum of squares.

It is challenging to observe the rank factorization of the simulated data on the cophenetic coefficient curve (Figure 4A). Moreover, there is no clue in deciding rank factorization simply by observing the cophenetic correlation (Figure 4A) and the RSS (Figure 4B) plots. Nevertheless, the UIK method successfully validated the results of Alexandrov et al [46] and calculated 10 mutational signatures for the simulated data. From the perspective of Frigyesi et al [11], Figure 4B further demonstrates that the actual optimal value of r=10 with the UIK method evaluates the ability of each value of the rank to classify the samples into the same number of classes, which could be smaller than the cophenetic measure (Figure 4A). Despite a decline in the cophenetic correlation coefficient value for r=5, 8, 10, the clusters are stationary and reflected as robust by Brunet et al [9], which produces unmeaningful results that match the actual signatures. Alexandrov et al [46] considered that the biological significance of the 10th cluster, for r=10, is less clear with the cophenetic measure. The sharp decrease in the cophenetic correlation coefficient at r=13 indicates that substantially less stability is achieved using more than 10 clusters. Since this approach does not always provide a clear and consistent cutoff for the choice of r, Alexandrov et al [46] utilized the average silhouette width of the *N* clusters as a measure of reproducibility for the whole solution. Here, the method of UIK estimation with the former results of actual signatures according to Alexandrov et al [46] was validated (see Supplementary Data S3-S4 in Multimedia Appendix 1).

## Discussion

### Principal Results

The novel finding of this study is the ability to apply the UIK method in selecting optimal ranks based on the RSS curve of factorization ranks of the NMF technique. First, this study employed the Golub et al [38] data set and simulated mutational process data [46,47] utilizing the UIK method, which does not require averaging out the results from different runs of the nmf() function [50] or considering the variance between each run.

In the second module, the UIK precisely estimates simulated data with known dimensions. The UIK technique is free of a priori rank parameter input and does not require setting initial parameters that considerably affect the performance. Finally, this method was tested on gene expression data deconvolution, achieving optimal rank estimation.

The proposed uikNMF technique was tested on both experimental gene expression and simulated mutational processes data sets. Moreover, our recent study of utilization of the UIK technique on NMF revealed the genetic links of type 2 diabetes (T2D) that could lead to the development of Alzheimer disease (AD) [51]. The study extracted the most significant genes, or so-called “metagenes,” using the elbow method in T2D data, which may be helpful for gaining insight into the mechanism of AD and the development of related therapeutics.

This study further shows that the UIK method provides a credible prediction for gene expression data and precisely estimates simulated data with known dimensions. The proposed UIK method based on the RSS curvature’s first inflection point to estimate the optimal rank is theoretically superior or equivalent to existing implementation and software. All the undertaking is done with R programming and is freely available.

As future work, some software functionality ideas include adapting the UIK method on NMF rank estimation in a single function package to accommodate analyses of gene expression, mutational processes, and other biological data sets at the molecular level.

### Limitations

The analysis has some limitations such that other NMF packages or software on gene expression research were not tested. This study demonstrates that the UIK method provides a credible prediction for gene expression data. However, it was simply assumed that the same algorithms of NMF are used, as far as the RSS and residual curves would be approximated the same way so that the UIK method would result in the same optimal ranks.

### Comparison With Prior Work

One of the arguments related to the choice of rank is to remove noise and recover the signatures [52]. However, when it comes to NMF, the choice of noise is not obvious as the noisy version of the target matrix must be nonnegative as well, which suggests that injected noise may also introduce bias [53]. In addition, the selection of the noise distribution is yet another hyperparameter that is not obvious to select. To handle the noise issue, it is suggested to use gene expression data sets (ie, microarrays) with low-quality reads and genes with a very low number of reads removed before DEGs analysis. The DEGs would then be used as the target matrix for the uikNMF method, as previously demonstrated with T2D gene expression data [51].

Several methods have been developed to select the optimal rank factorization [50]. For example, Brunet et al [9] proposed grabbing the first value of r for which the cophenetic coefficient rate was declining, whereas Frigyesi et al [11] pondered the minimum value at which the decrease in the RSS is lower than the decay of the RSS simulated from random data. The aim of this study was to develop a method for deciding how and which approach performs better on an estimation of the latent factors on given different algorithms of NMF.

### Conclusions

This study demonstrates that the elbow method provides a credible prediction for both gene expression data and for precisely estimating simulated mutational processes data with known dimensions. The suggested UIK method is faster than conventional methods with regard to usage of the consensus matrix as a benchmark for rank choice, while achieving considerably better computational adeptness without visual inspection on the curvatives. It is further argued that the suggested rank tuning method based on the elbow method with gene expression data is theoretically superior to the cophenetic measure. Lastly, the proposed method could be applied to other types of gene expression data sets to reveal the most significant genes (so-called “metagenes”) in various diseases, including T2D and other metabolic diseases, and may further be helpful for understanding the underlying mechanism of AD and related neurological disorders.

## Appendix

Multimedia Appendix 1 Alexandrov et al [46] simulated mutational signatures data summary (Supplementary Data S1). Implementation of the comparison of Gaujoux estimates of the esGolub subdata set with the unit invariant knee (UIK) method (Supplementary Data S2). The rank survey plot of Alexandrov et al [46] simulated mutational signatures data (Supplementary Data S3). Application of the UIK method on Alexandrov et al [46] simulated mutational signatures data (Supplementary Data S4).

## Abbreviations

AD: Alzheimer disease
ALL: acute lymphoblastic leukemia
DEG: differentially expressed gene
FA: factor analysis
MSE: mean squared error
NMF: nonnegative matrix factorization
PCA: principal component analysis
PRESS: predicted residual sum of squares
RSS: residual sum of squares
T2D: type 2 diabetes
UIK: unit invariant knee

## Data Availability

The Golub gene expression [38] and simulated mutational processes [46] data sets are publicly available. The data and related R studio codes supporting the findings of the article are available in Multimedia Appendix 1.
