# Supplementary material for: Decision of the Optimal Rank of a Nonnegative Matrix Factorization Model for Gene Expression Data Sets Utilizing the Unit Invariant Knee Method: Development and Evaluation of the Elbow Method for Rank Selection
Source: JMIR Bioinform Biotechnol. 2023 Jun 6;4:e43665. doi: 10.2196/43665 (PMC11135234; doi:10.2196/43665)
Supplement: Multimedia Appendix 1 [file bioinform_v4i1e43665_app1.pdf]

SUPPLEMANTARY DATA

The Decision of the Optimal Rank of a Nonnegative Matrix Factorization Model for Gene Expression Datasets Utilizing Unit Invariant Knee Method

Emine Güven[a]

[a]*Biomedical Engineering Department, Faculty of Engineering, Düzce University, Düzce, Turkey*

Supplementary Data S1

Alexandorav et al [46] the simulated mutational signatures data summary

simulated_data	list [5]	List of length 5
cancerType	character [1 x 1]	'WTSI BRCA Genomes'
originalGenomes	double [96 x 21]	31 34 9 21 13 15 110 91 9 87 100 46 122 112 13 107 52 ...
sampleNames	list [21]	List of length 21
subtypes	list [96]	List of length 96
types	list [96]	List of length 96

Supplementary Data S2

Implementation of the comparison of Gaujoux's estimates of the esGolub sub-data set as with the UIK method

```
require(NMF)
require(inflection)

data("esGolub")
sub_esGolub<-esGolub[1:200,]
estim.r<-nmfEstimateRank(sub_esGolub,r=2:6,nrun=10,seed=123456)
rankSurvey<-estim.r

pdf("plots/repeatEsgolub.pdf")
plot(rankSurvey)
dev.off()

x=rankSurvey$measures$rank
y=rankSurvey$measures$rss

rank=uik(x,y)

x1=min(x)
x2=max(x)
pdf("plots/uik_repeatEsgolub.pdf")
plot(x,y,type="b",xlab="factorization rank",ylab="RSS", pch=c(1,16,1,1,1),
                  xlim=c(2,6),font.lab=2,cex=1)
segments(rank,y[2],rank,y[5], lwd=2, pch=16,col="lightblue")
segments(x[1],y[2],x[2],y[2], lwd=2,pch=16, col="lightblue")

abline(v=rank,col='blue',lty=2)
axis(side=4,at=rank,labels=as.character(rank),cex.axis=0.6)
text(x = rank+0.2, y[2], label = "UIK",col="blue")
legend("topright",c("RSS","UIK, best rank"),cex=.8,
       col=c("black","black"),lty=c(1,NA),pch=c(1,16))

dev.off()
```

Supplemantary Data S3

The Rank survey plot of Alexandorav et al [46] simulated mutational signatures data

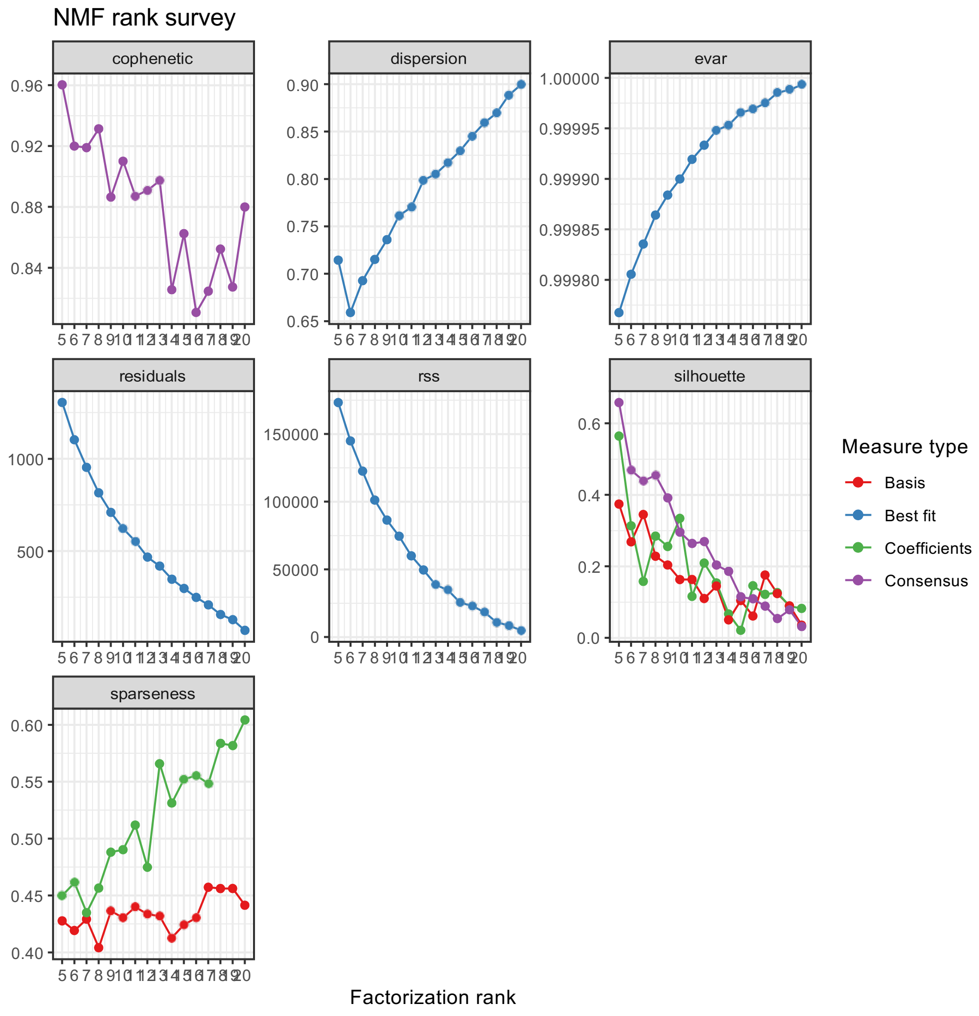

Supplementary Data S4

The UIK method on Alexandorav et al [46] simulated mutational signatures data

```
library(NMF)
```

Loading required package: pkgmaker
Loading required package: registry ## Loading
required package: rngtools ## Loading required
package: cluster

NMF - BioConductor layer [OK] | Shared memory capabilities [OK] | Cores 3/4

```
library(inflection) library(R.matlab)
```

R.matlab v3.6.2 (2018-09-26) successfully loaded. See ?R.matlab for help.
Attaching package: 'R.matlab'

The following objects are masked from 'package:base':
getOption, isOpen

```
data <- readMat('21_breast_WGS_substitutions.mat') summary(data)
```

```
##                     Length Class  Mode
## cancerType           1     -none- character ##
originalGenomes 2016   -none- numeric ##
sampleNames          21     -none- list
## subtypes             96     -none- list
## types                96     -none- list
```

```
str(data)
```

```
## List of 5
## $ cancerType      : chr [1, 1] "WTSI BRCA Genomes"
## $ originalGenomes: num [1:96, 1:21] 31 34 9 21 13 15 8 11 41 17... ## $
sampleNames        :List of 21
## ..$ :List of 1
## .. ..$ : chr [1, 1] "PD3851a"
##   ..$ :List of 1
##   .. ..$ : chr [1, 1] "PD3890a"
##   ..$ :List of 1
##   .. ..$ : chr [1, 1] "PD3904a"
##   ..$ :List of 1
##   .. ..$ : chr [1, 1] "PD3905a"
##   ..$ :List of 1
##   .. ..$ : chr [1, 1] "PD3945a"
##   ..$ :List of 1
##   .. ..$ : chr [1, 1] "PD4005a"
##   ..$ :List of 1
##   .. ..$ : chr [1, 1] "PD4006a"
##   ..$ :List of 1
##   .. ..$ : chr [1, 1] "PD4085a"
##   ..$ :List of 1
##   .. ..$ : chr [1, 1] "PD4086a"
##   ..$ :List of 1
```

```
## .. ..$ : chr [1, 1] "C>T"
## ..$ :List of 1
## .. ..$ : chr [1, 1] "C>T"
## ..$ :List of 1
## .. ..$ : chr [1, 1] "C>T"
## ..$ :List of 1
## .. ..$ : chr [1, 1] "C>T"
## ..$ :List of 1
## .. ..$ : chr [1, 1] "T>A"
## ..$ :List of 1
## .. ..$ : chr [1, 1] "T>A"
## ..$ :List of 1
## .. ..$ : chr [1, 1] "T>A"
## ..$ :List of 1
## .. ..$ : chr [1, 1] "T>A"
## ..$ :List of 1
## .. ..$ : chr [1, 1] "T>C"
## ..$ :List of 1
## .. ..$ : chr [1, 1] "T>C"
## ..$ :List of 1
## .. ..$ : chr [1, 1] "T>C"
## ..$ :List of 1
## .. ..$ : chr [1, 1] "T>C"
## ..$ :List of 1
## .. ..$ : chr [1, 1] "T>G"
## ..$ :List of 1
## .. ..$ : chr [1, 1] "T>G"
## ..$ :List of 1
## .. ..$ : chr [1, 1] "T>G"
## ..$ :List of 1
## .. ..$ : chr [1, 1] "T>G"
## - attr(*, "header")=List of 3
## ..$ description: chr "MATLAB 5.0 MAT-file, Platform: MACI64, Created on: Thu Feb 15 20:52:022018 ## ..$ version : chr "5"
## ..$ endian : chr "little"
```

```r
originalGenome<-data$originalGenomes
head(originalGenome)
```

```
##       [,1] [,2] [,3] [,4] [,5] [,6] [,7] [,8] [,9] [,10] [,11] [,12] [,13] [,14]
## [1,]    31  110  122   94  243   74  198   61   31    34   112   210   122   228
## [2,]    34   91  112   69  163   66  173   51   19    18    50   176   133   169
## [3,]     9    9   13   11   24   12   33    7    8     3     7    33    12    21
## [4,]    21   87  107   65  155   64  192   49   23    26    44   176    96   158
## [5,]    13  100   52   66  130   56  164   18   18    15    30   126    95   128
## [6,]    15   46   42   41   78   34   96   18   14    11    28    85    72    74
##      [,15] [,16] [,17] [,18] [,19] [,20] [,21]
## [1,]   128   165    48    28    58    58    64
## [2,]   138    55    42    24    72    36    43
## [3,]    15    20    16     6    12    13     7
## [4,]   142    65    40    13    45    37    35
## [5,]   102   191    60     8    37    23    25
## [6,]    71    72    26     5    27    19    18
```

```
### nmf rank suvey
simulated.r<-nmfEstimateRank(originalGenome,r=2:21,nrun=35,seed=123456)

#simulated.r2<-nmfEstimateRank(originalGenome,r=5:20,nrun=30,seed=123456)
rankSurvey<-simulated.r
#rankSurvey<-simulated.r2

#pdf("plots/repeatEsgolub.pdf")
plot(rankSurvey)
```

Warning: Removed 1 row(s) containing missing values (geom_path). ## Warning:

Removed 1 rows containing missing values (geom_point).

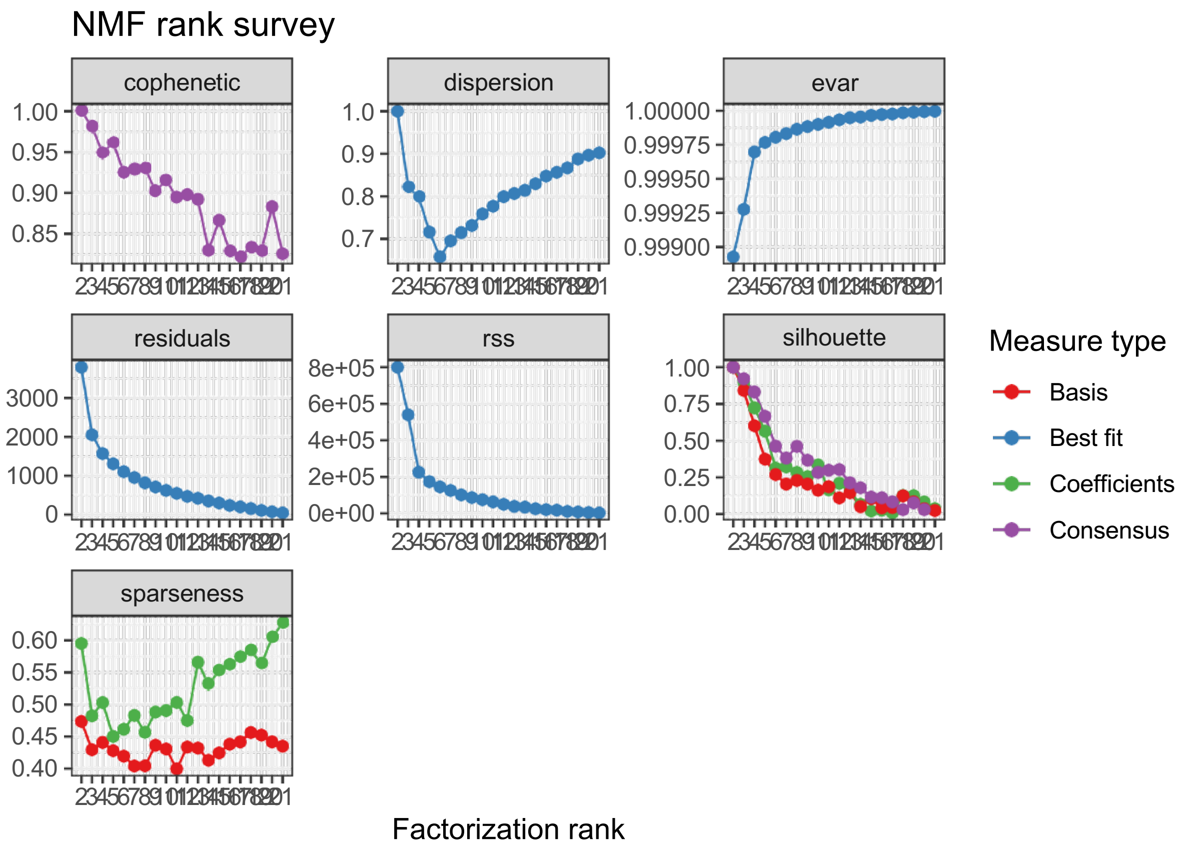


```
#dev.off()
```

```
x=rankSurvey$measures$rank
y=rankSurvey$measures$rss

rank=uik(x,y) rank=10

#plot(x,y,pch=19,cex=0.9)
x1=min(x) x2=max(x)
#pdf("plots/uik_repeatEsgolub.pdf")
plot(x,y,type="b",xlab="factorization rank",ylab="RSS",pch=c(rep(1,8),16,rep(1,11)),xlim=c(5,21),font.l
```

```
#par(new=TRUE)
#plot(rank,y[4],pch=16,col="blue", xaxt='n', yaxt='n',ann=FALSE) segments(rank,y[9],rank,y[20], lwd=2,
pch=16,col="lightblue") segments(x[1],y[9],x[9],y[9], lwd=2,pch=16, col="lightblue")

abline(v=rank,col='blue',lty=2)
axis(side=4,at=rank,labels=as.character(rank),cex.axis=0.6) text(x = rank+0.4, y[7],
label = "UIK",col="blue")
#srt = 90,col='navyblue') # Rotation

legend("topright",c("RSS","UIK, best rank"),cex=.8,
           col=c("black","black"),lty=c(1,NA),pch=c(1,16))
```

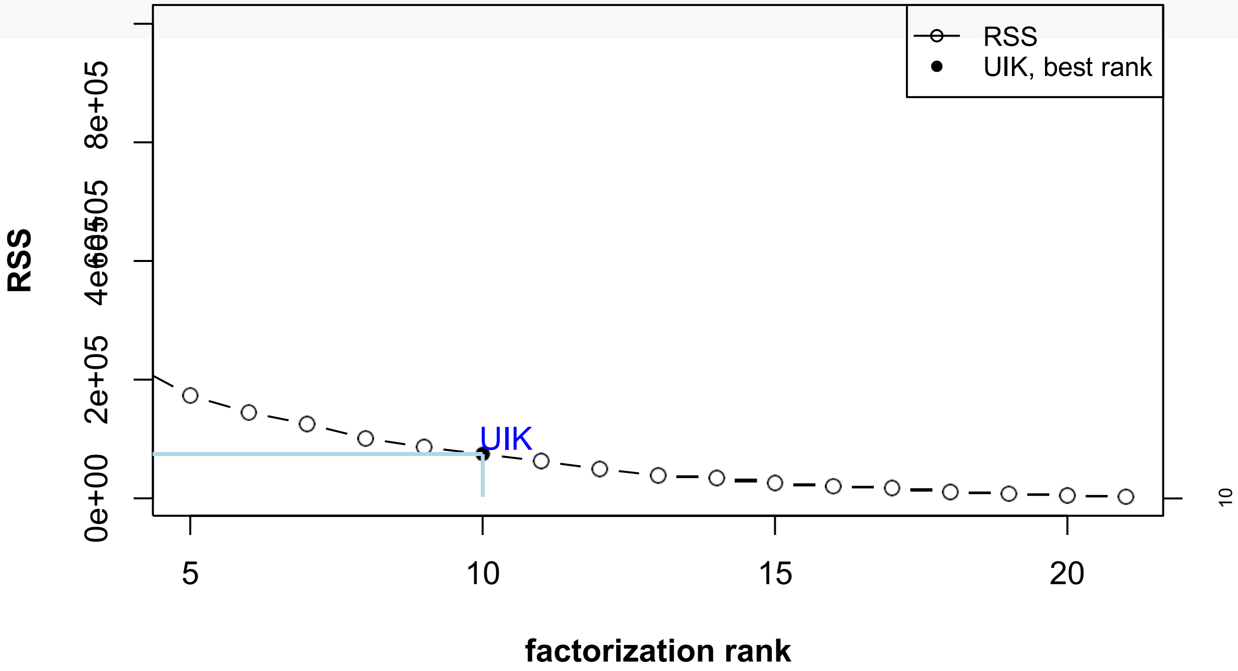


```
#dev.off()
```
